# RNA-Sequencing Characterization of lncRNA and mRNA Functions in Septic Pig Liver Injury

**DOI:** 10.3390/genes14040945

**Published:** 2023-04-20

**Authors:** Jing Zhang, Zhihui Xue, Qingbo Zhao, Keke Zhang, Ao Zhou, Liangyu Shi, Yulan Liu

**Affiliations:** Hubei Key Laboratory of Animal Nutrition and Feed Science, Hubei Provincial Center of Technology Innovation for Domestic Animal Breeding, Wuhan Polytechnic University, Wuhan 430023, China; judyzhang1103@126.com (J.Z.); xzh13503975007@126.com (Z.X.); zhqb0416@126.com (Q.Z.); zhangkk23151979@126.com (K.Z.); zhouao2008@aliyun.com (A.Z.); liangyu_shi@whpu.edu.cn (L.S.)

**Keywords:** lipopolysaccharide, pigs, liver injury, lncRNAs, transcriptomics

## Abstract

We assessed differentially expressed (DE) mRNAs and lncRNAs in the liver of septic pigs to explore the key factors regulating lipopolysaccharide (LPS)-induced liver injury. We identified 543 DE lncRNAs and 3642 DE mRNAs responsive to LPS. Functional enrichment analysis revealed the DE mRNAs were involved in liver metabolism and other pathways related to inflammation and apoptosis. We also found significantly upregulated endoplasmic reticulum stress (ERS)-associated genes, including the receptor protein kinase receptor-like endoplasmic reticulum kinase (PERK), the eukaryotic translation initiation factor 2α (EIF2S1), the transcription factor C/EBP homologous protein (CHOP), and activating transcription factor 4 (ATF4). In addition, we predicted 247 differentially expressed target genes (DETG) of DE lncRNAs. The analysis of protein-protein interactions (PPI) and Kyoto Encyclopedia of Genes and Genomes (KEGG) pathway detected key DETGs that are involved in metabolic pathways, such as N-Acetylgalactosaminyltransferase 2 (GALNT2), argininosuccinate synthetase 1 (ASS1), and fructose 1,6-bisphosphatase 1 (FBP1). LNC_003307 was the most abundant DE lncRNA in the pig liver, with a marked upregulation of >10-fold after LPS stimulation. We identified three transcripts for this gene using the rapid amplification of the cDNA ends (RACE) technique and obtained the shortest transcript sequence. This gene likely derives from the nicotinamide N-methyltransferase (*NNMT*) gene in pigs. According to the identified DETGs of LNC_003307, we hypothesize that this gene regulates inflammation and endoplasmic reticulum stress in LPS-induced liver damage in pigs. This study provides a transcriptomic reference for further understanding of the regulatory mechanisms underlying septic hepatic injury.

## 1. Introduction

Endotoxin is a lipopolysaccharide (LPS) component on the outer membrane of gram-negative bacteria [1] and an important pathogenic factor causing systemic inflammatory reactions in pigs, with potentially severe health problems. The liver is one of the most important organs in the body, responsible for detoxification, digestion, metabolism, and immunity [2]. In addition, the liver clears LPS from the body, whereby it is a primary target for LPS-induced inflammatory molecules that can cause liver damage [3]. Liver dysfunction can in turn result in growth issues, metabolic disorders, and death in pigs.

Long non-coding RNAs (lncRNAs) are usually longer than 200 nucleotides and expressed in most tissues in different organisms [4]. Several forms of liver injury have been previously linked to lncRNAs, including nonalcoholic liver disease, cholestatic liver injury, and liver ischemia-reperfusion [5,6,7]. In sepsis-induced liver injury, the LncRNA NEAT1 regulates the Let-7a/Toll-like receptor 4 (TLR4) axis to promote inflammation [8], while lncRNA MALAT1 deficiency attenuates interleukin-1β (IL-1β) and tumor necrosis factor-alpha (TNF-α) production during live injury [9], and lncRNA X inactivate-specific transcript (XIST) silencing ameliorates sepsis-induced acute liver injury by downregulating the expression of bromodomain-containing protein 4 (BRD4) [10]. 

LPS induces acute liver injury through inflammation and oxidative stress and is an important initiator of sepsis [11,12]. Previous studies have used a septic animal model induced by LPS to evaluate mechanisms and therapeutic strategies for sepsis-induced liver injury [13]. While the role of lncRNAs during liver disease has been extensively studied in humans, mice, and rats, little is currently known about their functional importance during LPS-induced hepatic injury in pigs, which are an ideal model species for studying human disease due to similar organ size and physiology [14]. Previously, we successfully established a sepsis model in pigs using LPS injection and induced severe hepatic injury in the animal models [15,16]. Here, we investigate lncRNAs and mRNAs associated with LPS-induced liver damage in pigs using high-throughput RNA-sequencing and provide valuable new information for an in-depth understanding of the transcriptome and the underlying mechanisms of LPS-induced liver injury in pigs.

## 2. Materials and Methods

### 2.1. Tissues Samples

All procedures were approved by the Animal Care and Use Committee of Wuhan Polytechnic University (Wuhan, China). The liver tissue samples of septic pigs used for lncRNA-sequencing were collected in our previous work and stored in the laboratory. Briefly, a total of twelve weaned piglets (Duroc × Large White × Landrace, 28 ± 3 d) with body weights (BW) of 7.02 ± 0.21 kg were randomly divided into two groups (six animals per treatment). After injection with LPS (*Escherichia coli* serotype 055:B5; 100 μg/kg body weight) or 0.9% sterile saline solution for 4 h, the piglets were sacrificed and their livers harvested [17].

In addition, the tissue samples used to validate the expression of the lncRNA and analyze its tissue expression patterns were collected in the previous work [16] and stored in the laboratory. Briefly, a total of 42 weaned piglets (Duroc × Large White × Landrace, 28 ± 3 d) with body weights (BW) 7.1 ± 0.9 kg were randomly divided across seven treatments (six animals per treatment). The piglets were sacrificed at 0 h (pre-LPS challenge), 1, 2, 4, 8, 12, and 24 h (post-LPS challenge) after LPS injection. Various tissue samples (heart, lung, skeletal muscle, spleen, liver, stomach, kidney, jejunum, brain and thymus) were dissected and snap-frozen in liquid nitrogen [16].

### 2.2. High-Throughput Sequencing

Total RNA was extracted from the liver using Trizol (Invitrogen, Waltham, MA, USA). Qualified RNA from the Saline and LPS-treated groups was used for lncRNA-sequencing. The sequencing libraries were constructed according to the manual and then used for RNA-sequencing on an Illumina HiSeq 3000 (Shanghai Genergy, Shanghai, China). We filtered the raw data by removing low-quality reads and adapters. The obtained high-quality clean reads were then mapped to the pig reference genome (Sus scrofa 11.1) using HISAT2 (v2.0.4). The assembly of mapped reads into transcripts was done using Cufflinks (v.2.1.1). After excluding transcripts ≤ 200 bp or with an fragment per kilobase of transcript per million mapped reads (FPKM) < 0.5 in one group, we merged the remaining transcripts using Cuffmerge (v.2.1.1). The unannotated transcripts were kept as putatively lncRNA transcripts and their coding potential predicted by Coding-Non-Coding-Index (CNCI), Coding Potential Calculator (CPC2), and PfamScan (v1.3). After removing transcripts with coding capacities, the remaining transcripts were identified to be lnc RNAs. The RNA-seq data were deposited in NCBI: https://www.ncbi.nlm.nih.gov/sra/PRJNA908120 (accessed on 31 January 2023).

### 2.3. Analysis of Differentially Expressed (DE) LncRNAs and mRNAs

FPKM was used to normalize the expression of lncRNAs and mRNAs in the control and LPS groups. The criteria used to establish significantly different expressions were |log2 Fold Chang (FC)| ≥ 1.0 and *q*-value < 0.05.

### 2.4. Functional Enrichment Analysis

The DE mRNAs were subjected to functional enrichment analyses using OmicShare (https://www.omicshare.com/tools (accessed on 1 March 2023)) [18]. A significant enrichment was considered for Gene Ontology (GO) terms and Kyoto Encyclopedia of Genes and Genomes (KEGG) pathways with a *p*-value < 0.05.

Functional enrichment of the co-expressed DE mRNAs was analyzed with Cytoscape ClueGO (v2.5.9), a useful plug-in tool available in Cytoscape that performs enrichment analysis by visualizing the functional ontology of target genes and pathway annotation networks [19].

### 2.5. The cis- and Trans-Target Gene Prediction of DE lncRNAs

To explore their possible functions, we predicted cis target genes for lncRNAs located 100kb upstream and downstream of each lncRNA. We calculated Pearson’s correlation coefficients (PCCs) between mRNAs and lncRNAs using the R-Hmisc package. LncRNA-mRNA pairs with |PCC| > 0.9 and *p* < 0.05 were considered to have a target relationship [20].

### 2.6. Integration of Protein-Protein Interaction Networks

The Search Tool for the Retrieval of Interacting Genes (STRING) is a biological database for predicting protein-protein interaction (PPI) analysis. We used Cytoscape’s (v3.9.1) STRING app [21] to construct the PPI network with a confidence score > 0.4 and string enrichment analysis with a 0.05 cutoff.

### 2.7. Quantitative Real-Time Polymerase Chain Reaction (qRT-PCR)

cDNA synthesis and qRT-PCR were carried out as previously described [15]. Gene expression was analyzed using the Applied Biosystems 7500 real-time PCR system (Applied Biosystems, Waltham, MA, USA), using β-actin as an internal reference. Data analysis was performed via the 2^−△△CT^ method [22]. The specific primers are shown in Table S1.

### 2.8. 5′ and 3′ Rapid Amplification of cDNA Ends (RACE)

The RACE technique was used to determine lncRNA initiation and termination sites from the total RNA extracted from pig livers. Next, 5′-RACE analyses were performed using a SMARTer RACE cDNA amplification kit (Takara Bio USA, Inc., San Jose, CA, USA) according to the manufacturer’s instructions. The gene-specific primers (GSP) are listed in Supplementary Table S1. For 3′-RACE analysis, nested PCR was carried out with 3′ GSP1 (External) and 3′ GSP2 (Internal) according to the manufacturer’s instructions. GSPs are also listed in Table S1.

### 2.9. Statistical Analysis

The data are presented as mean ± standard errors of the mean (SEM). Differences were tested using ANOVA and Student’s paired *t*-test. Statistical significance was defined as *p* < 0.05.

## 3. Results

### 3.1. Overview of RNA-Sequencing Data

After performing quality controls, we obtained the following numbers of clean reads with Q3 > 94% in each library: N1 (101,547,492), N2 (150,724,416), N3 (110,219,924), L1 (127,916,070), L2 (161,346,466), and L3 (137,305,488) (Table 1). Of these, a total of 83.01% (N1), 82.45% (N2), 82.00% (N3), 80.96% (L1), 80.51% (L2), and 82.58% (L3) reads from the six libraries were mapped to the pig reference genome (Sus scrofa 11.1).

### 3.2. LncRNA and mRNA Expression Profiles

**Figure 1:**
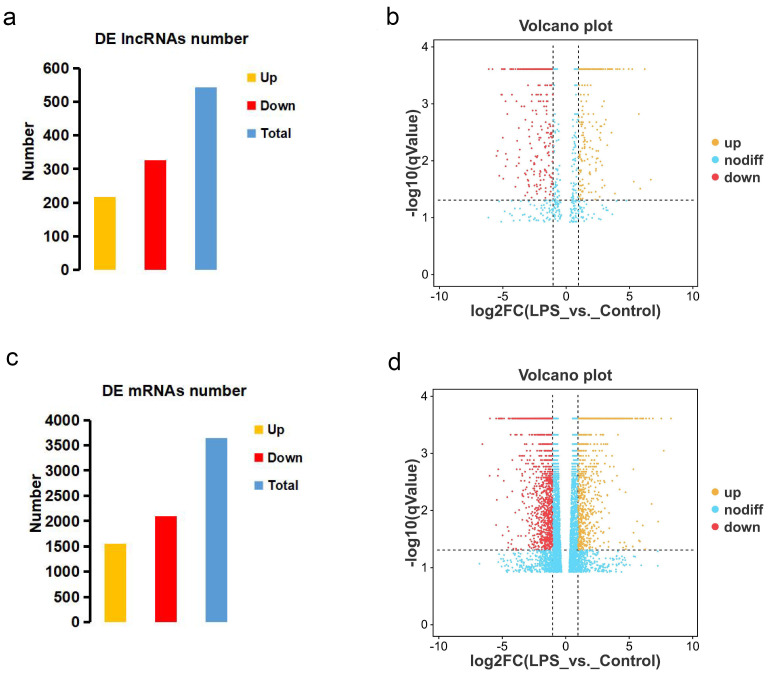
Variation in the expression of lncRNAs and mRNAs in liver tissues after LPS challenge, including DE lncRNAs (**a**) and DE mRNAs (**c**). Volcano plot of DE lncRNAs (**b**) and DE mRNAs (**d**). The representations are as follows: the x-axis shows log2 FC, while the y-axis shows −log10 of the *q*-value. The RNAs with *q*-values < 0.05 and log2FC ≥ 1 are shown as yellow dots; the RNAs with *q*-values < 0.05 and log2FC ≤ −1 are shown as red dots.

According to the sequencing data, a total of 543 DE lncRNAs were identified in the liver tissues after the LPS challenge, of which 217 were upregulated and 326 downregulated (Figure 1a, Supplementary Table S2). In addition, a total of 3642 DE mRNAs were identified in the liver after the LPS challenge, of which 1553 were upregulated and 2089 downregulated (Figure 1c, Supplementary Table S2). DE lncRNAs and DE mRNAs were mapped in a volcano plot (Figure 1b,d).

**Figure 2:**
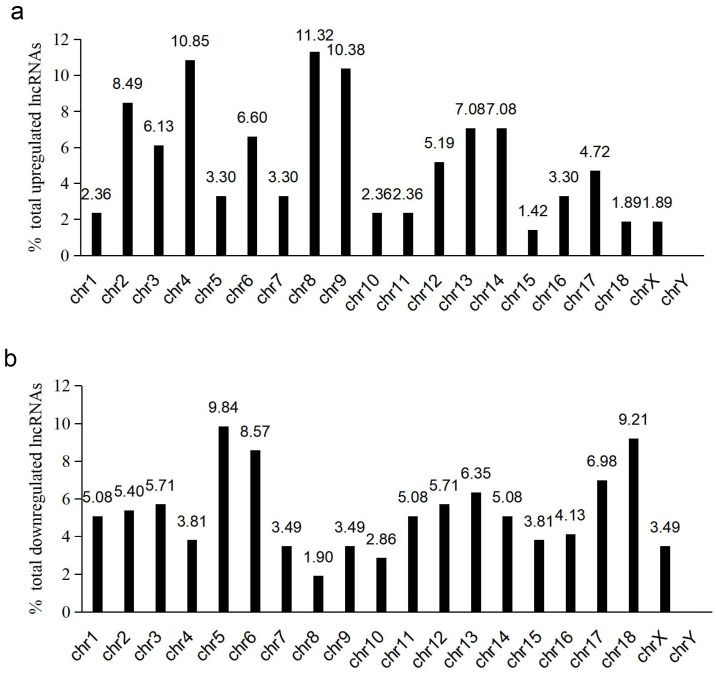
Chromosomal distribution of DE lncRNAs. The upregulated (**a**) and downregulated (**b**) lncRNAs were widely distributed across all chromosomes except for chromosome Y.

Among the 217 upregulated lncRNAs, most were located on chromosomes 8 (11.32%), 4 (10.85%) and 9 (10.38%), with chromosomes 15 (1.42%), 18 (1.89%), X (1.89%) and Y (0.00%) having a proportion lower than 2% (Figure 2a). The 326 downregulated lncRNAs were mainly distributed on chromosomes 5 (9.84%) and 18 (9.21%), with chromosomes 8 (1.90%) and Y (0.00%) containing a proportion lower than 2% (Figure 2b).

### 3.3. Function and Pathway Enrichment Analyses of DE mRNAs

**Figure 3:**
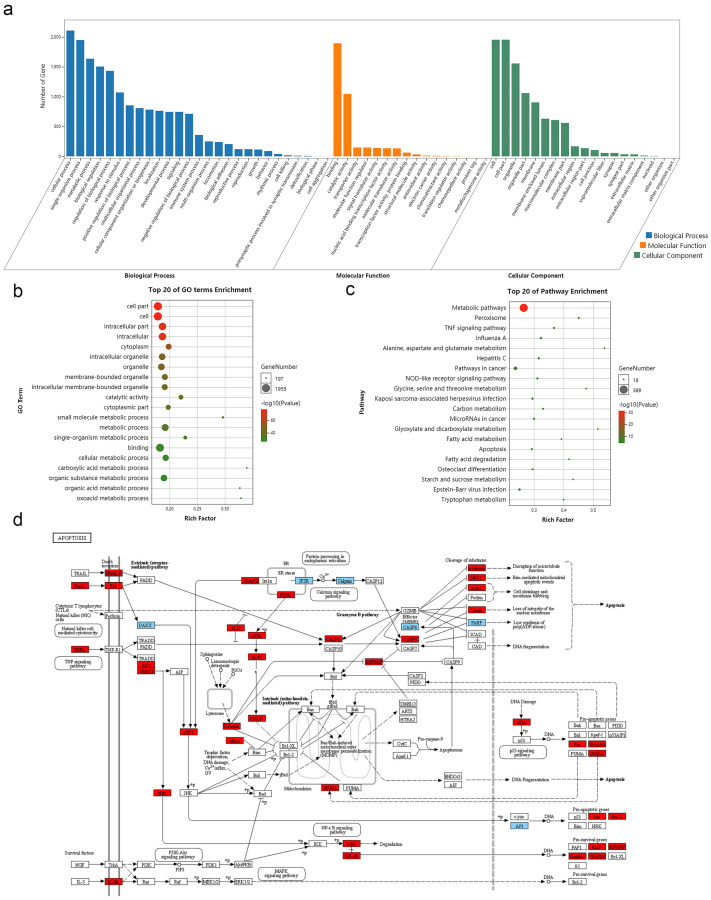
GO and KEGG pathway analysis of the DE mRNAs. (**a**) The DEGs were classified into three main categories: molecular function, cellular component, and biological process. (**b**) The top 20 significantly enriched GO-BP terms. (**c**) The top 20 significantly enriched KEGG pathways. The color represents the *p*-value, and the size of the dot denotes the number of genes involved in the pathway. (**d**) The DEGs associated with Apoptosis pathway. The red and blue boxes represent upregulated and downregulated genes, respectively.

Go functional classification was performed to evaluate the possible function of the DE mRNAs, which were classified into 3 categories, including 16 molecular function (MF) terms, 19 cellular component (CC) terms, and 27 biological process (BP) terms (Figure 3a). Figure 3b shows the top 20 significantly enriched GO terms of biological processes. We found that these differentially expressed genes (DEGs) are closely related to metabolic processes, such as the small molecule metabolic process, carboxylic acid catabolic process, organic acid catabolic process, oxoacid metabolic process, and catabolic process.

Figure 3c shows the top 20 enriched pathways associated with DE mRNAs. KEGG analysis also revealed these DE mRNAs are significantly involved in metabolic-related pathways, such as Amino acid metabolism, Carbon metabolism, Glyoxylate and dicarboxylate metabolism, Fatty acid metabolism, Starch and sucrose metabolism, and Tryptophan metabolism. In addition, inflammation-related pathways are also significantly enriched, including the TNF signaling pathway, NOD-like receptor signaling pathway, and apoptosis. We observed upregulation of a few differentially expressed genes in the liver related to apoptosis pathways, as shown in Figure 3d. These genes include Caspase 8 (Casp8) and Caspase 3 (Casp3), which are crucial mediators of apoptosis.

### 3.4. Target Gene Prediction of DE lncRNAs

**Figure 4:**
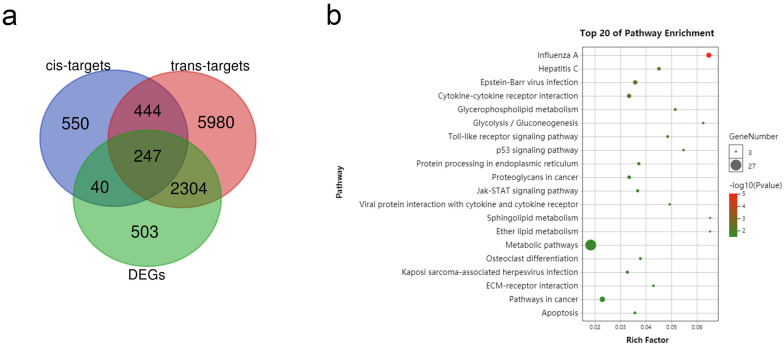
Functional analysis of DETGs. (**a**) Venn diagram of target genes of DE lncRNAs and DEGs. (**b**) The top 20 significantly enriched KEGG pathways of the 247 overlapping DETGs.

LncRNAs can perform gene both cis and trans-gene regulation. The predicted cis- and trans-targets of DE lncRNAs are listed in Supplementary Table S3. The target genes of DE lncRNAs and DE mRNAs were intersected, allowing us to identify 247 differentially expressed target genes (DETGs) that overlap (Figure 4a). KEGG pathway enrichment indicated that these DETGs are involved in the metabolic pathways, Toll-like receptor signaling pathway, Jak-STAT signaling pathway and Apoptosis (Figure 4b).

### 3.5. PPI Network Construction and Selection of Hub Genes

**Figure 5:**
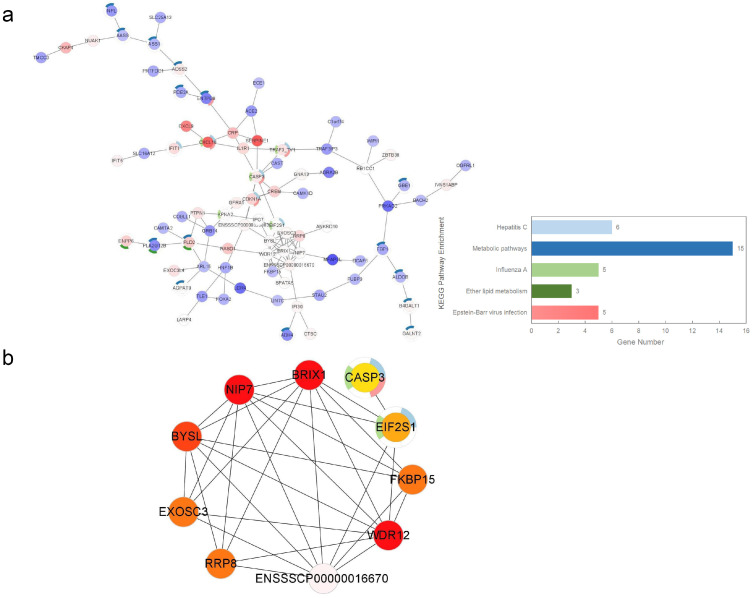
Identification of hub genes from the PPI network with CytoHubba. (**a**) The PPI network of the DETGs. Up- and downregulated genes are shown in red and blue, respectively. The darker the color, the greater the fold change observed. (**b**) The top ten hub genes were ranked using the MCC score.

We imported 247 DETGs into the string database and constructed a PPI network using Cytoscape. According to PPI interaction network and KEGG pathway enrichment analyses (Figure 5a), we found five upregulated DETGs (*ADSS2*, *PLD2*, *B4GALT1*, *GALNT2*, *AGPAT9*) and ten downregulated DETGs (*ALDOB*, *ASS1*, *ENTPD8*, *ADH4*, *PLA2G12B*, *FBP1*, *GBE1*, *AASS*, *PDE2A*, *NPL*) that are related to the metabolic pathways. In addition, the six upregulated DETGs are involved in Hepatitis C, including *CDKN1A*, *TRAF3*, *CXCL10*, *CASP3*, *EIF2S1*, and *IFIT1*. The top 10 essential nodes ranked by Maximal Clique Centrality (MCC) were selected, including the genes *BRIX1*, *WDR12*, *NIP7*, *BYSL*, *EXOSC3*, *RRP8*, *CASP3*, and *EIF2S1*. The result is shown in Figure 5b.

### 3.6. qPCR Validation

**Figure 6:**
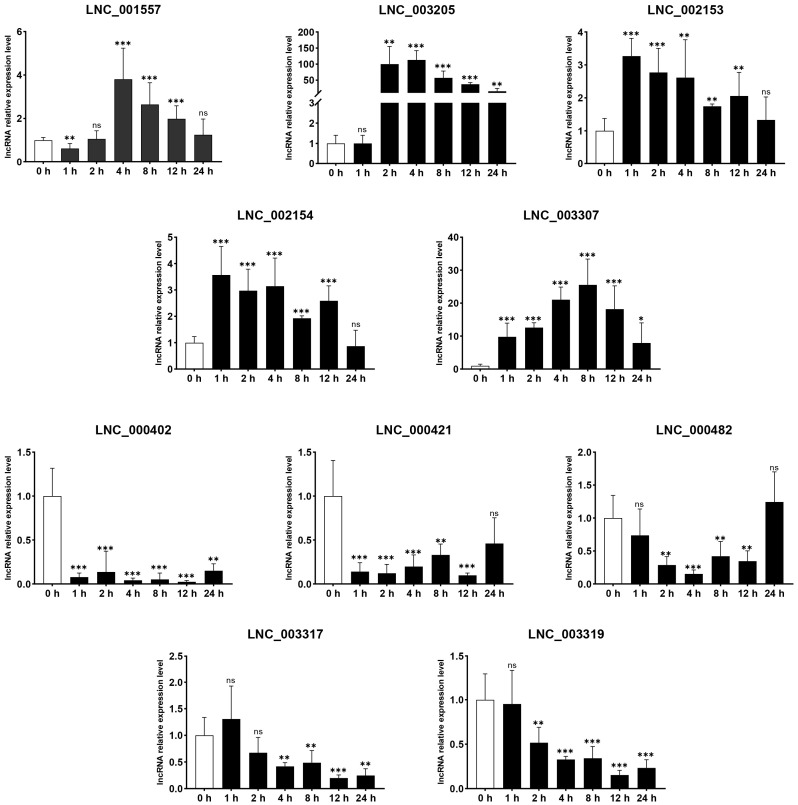
qPCR validations of the five upregulated and five downregulated lncRNAs in pig livers at 0, 1, 2, 4, 8, 12, and 24 h following LPS challenge. The data represent the mean ± SEM. *n* = 6. * *p* < 0.05; ** *p* < 0.01; *** *p* < 0.001 vs. 0 h; ns: no statistical difference.

In this study, we evaluated the expression of lncRNAs and mRNAs in pig livers challenged with LPS for 4 h using sequencing. To validate the reliability of the sequencing results, we selected ten DE lncRNAs and examined their expression changes in pig livers at 0, 1, 2, 4, 8, 12, and 24 h after LPS challenge using qPCR. As shown in Figure 6, the expressions of LNC_001557, LNC_003205, LNC_002153, LNC_002154, and LNC_003307 were significantly upregulated 4h after LPS challenge; while the expressions of LNC_000402, LNC_000421, LNC_000482, LNC_003317 and LNC_003319 were significantly downregulated around the same time. These observations are consistent with the sequencing results. Notably, the expression of LNC_003205 was markedly upregulated (>50-fold) at 2 h to 4 h following LPS stimulation, while LNC_003307 was upregulated (>10-fold) at 2 h to 12 h. 

### 3.7. Identification and Characterization of LNC_003307

**Figure 7:**
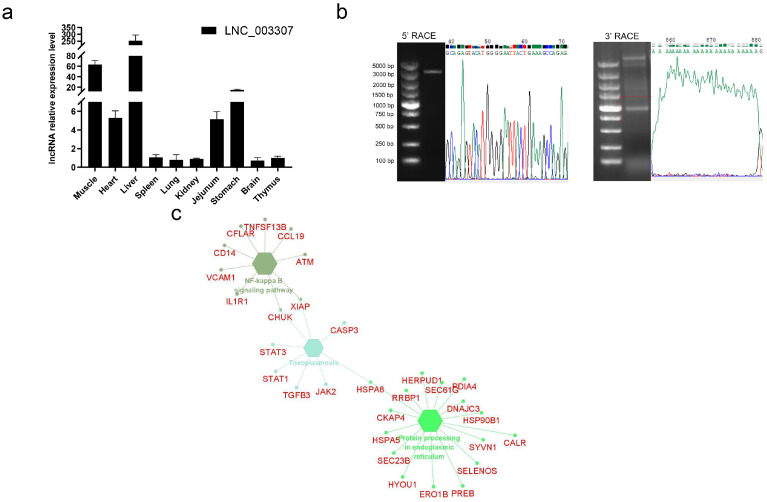
Identification and characterization of LNC_003307. (**a**) Expression patterns of LNC_003307 across various pig tissues. The data represent the mean ± SEM; *n* = 3. (**b**) The full-length sequence of LNC_003307 was obtained by RACE analysis. Agarose gel electrophoresis of the PCR product from the 5′-RACE and 3′-RACE procedure. (**c**) KEGG enrichment of LNC_003307 DETGs using Cytoscape GlueGO.

We analyzed the top 5 DE lncRNAs expressed in pig liver tissues and found that LNC_003307 was the most abundant DE lncRNA in our study (Table 2). Afterward, we measured tissue expression patterns of LNC_003307. As shown in Figure 7a, the expression level of LNC_003307 was higher in the liver and muscle compared to other tissues. RACE was further employed to determine the transcriptional start and termination sites of LNC_003307. We successfully obtained the sequence of one LNC_003307 transcript (Figure 7b and File S1). The transcription direction of LNC_003307 was similar to that of the nicotinamide N-methyltransferase (*NNMT*) derived from Exons 1 and 2 and Intron 2 of the *NNMT* gene. To explore the potential functions of LNC_003307, we performed KEGG pathway analysis of its DETGs using Cytoscape GlueGO. The DETGs of LNC_003307 were enriched for three KEEG pathways, including the NF-κB signaling pathway and protein processing in the endoplasmic reticulum (Figure 7c).

## 4. Discussion

LncRNAs are largely responsible for the biological processes behind the pathophysiology of liver disease [23]. However, the molecular mechanisms of associated lncRNAs responsible for LPS-induced liver injury remain largely unknown. In a previous study, Yang et al. [17] applied high-thought sequencing to profile global miRNA expression changes in piglet livera after the LPS challenge. However, no systematic identification of gross lncRNAs associated with LPS-induced liver damage was reported. Here, we present the first lncRNA transcriptome profile of liver injury in a pig model of sepsis. The identification of 543 DE lncRNAs and 3642 DE mRNAs revealed a higher number of dysregulated mRNAs in response to LPS.

LPS can induce hepatic morphological damage in piglets after challenge for 4 h, according to our previous study [15,16,17]. Here, we found that apoptosis-related genes were significantly upregulated, suggesting that LPS-induced liver injury was closely associated with apoptosis. TNF protein superfamily members, including TNF-a, Fas cell surface death receptor ligand (FASL), and TNF-associated apoptosis-inducing ligand (TRAIL), are the main inducers of hepatocyte death. The TNF-a/tumor necrosis factor receptor (TNFR1), FAS/FASL, and TRAIL/TRAILR pathways can activate CASP8 and then initiate a proteolytic cascade that involves CASP3, CASP6, and CASP7, which ultimately leads to hepatocyte apoptosis [24,25,26]. We showed that TRAILR, FAS, CASP8, and CASP3 were upregulated in the LPS-stimulated piglet livers, and that the predicted lncRNA target genes were also enriched in the apoptosis pathway. Interesting, endoplasmic reticulum stress (ERS) markers were upregulated in pig livers following the LPS challenge, including the receptor protein kinase receptor-like endoplasmic reticulum kinase (PERK), eukaryotic translation initiation factor 2α (eIF2α, also known as *EIF2S1*), the transcription factor C/EBP homologous protein (CHOP), and activating transcription factor 4 (ATF4). Wang et al. [27] demonstrated that acute ERS induces inflammation, complement activation, and lipid metabolism disorder, leading to liver injury in pigs. In human mesangial cells, anti-dsDNA antibodies induced the activation of NF-κB and upregulated the expression of IL-1β and TNF-α, depending on the PERK/ATF4/NF-κB pathway [28]. Accordingly, our result suggest that ERS might be induced in the pig liver by LPS, leading to the accentuation of inflammation and liver injury mediated via the PERK/eIF2α/ATF4 pathway.

Liver diseases are closely associated with metabolic syndromes [29]. In this study, GO and KEGG functional enrichment analysis showed that DE mRNAs were majorly implicated in the metabolic process, including Amino acid metabolism, Carbon metabolism, Fatty acid metabolism, and Tryptophan metabolism, all of which might be responsible for LPS-induced metabolic syndrome observed during septic liver injury. We identified a total of 247 DETGs among DE lncRNAs, some of which were also involved in the metabolic pathways. The PPI network analysis identified some DETGs that might play key roles in the metabolic pathways related to liver injury. For example, we found that N-Acetylgalactosaminyltransferase 2 (*GALNT2*) expression was significantly increased, which might be beneficial for inhibiting TNF-α release and inflammation. GALNT2 is an enzyme regulating the initial step of mucin O-glycosylation and is involved in lipid metabolism, insulin sensitivity, and inflammation [30]. LPS elevates TNF-α plasma levels in GALNT2^-/-^ KO mice, confirming GALNT2 participates in TNF-α release and regulation [31]. Another example is that of argininosuccinate synthetase 1 (ASS1), a rate-limiting enzyme in arginine metabolism that is downregulated in a Thioacetamide (TAA)-induced liver injury model [32]. TAA triggers the onset of acute liver inflammation and oxidative stress by mimicking the pathological process of liver injury [33]. The activation of the Farnesoid X receptor (FXR), an upstream transcription factor of ASS1, can directly promote ASS1 transcription and enhance arginine synthesis, leading to the alleviation of TAA-mediated liver injury [32]. Recent studies demonstrated the protective effects of L-arginine in hepatic injury animal models [34,35,36]. In our study, we found that *ASS1* was significantly downregulated in pig livers after LPS challenge, which might inhibit arginine synthesis and aggravate septic liver injure. In addition, we found that the expression of fructose 1,6-bisphosphatase 1 (*FBP1*) was significantly decreased in the liver of septic pigs. We note that FBP1 is a rate-limiting gluconeogenic enzyme whose depletion in the hepatocytes disrupts liver lipid metabolism and might cause ERS [37]. Phosphodiesterase 2 (PDE2A) is a regulator of cAMP/cGMP levels, mitochondria function, and protein phosphorylation [38,39,40] that is crucial for mouse liver development and hematopoiesis [41]. Rentsendorj et al. [42] found that PDE2A was increased in LPS-treated mouse alveolar macrophages negatively regulating the expression of iNOS after LPS exposure. However, we found that *PDE2A* expression was significantly decreased in LPS-treated pig liver, which might aggravate LPS-induced iNOS expression and liver injury. In addition, we selected a total of 10 hub genes from the PPI network, of which those with the highest scores were *NIP7*, *BRIX1*, and *WDR12*, all related to ribosome biogenesis [43,44,45].

LNC_003307 was the most abundant DE lncRNA in the pig liver and upregulated by >10-fold after LPS stimulation. We identified three transcripts in LNC_003307 using 3′-and 5′-RACE methods. We successfully retrieved the sequence of the shortest transcript and found it derived from the pig *NNMT* gene, which was also significantly upregulated by LPS stimulation in pig liver according to our sequencing data. We note that NNMT is an important enzyme that regulates metabolism, and that ERS-induced upregulation of NNMT promotes the development of alcohol-related fatty liver [46]. Functional analysis showed LNC_003307 DETGs were enriched in the NF-κB signaling pathway and protein processing in endoplasmic reticulum. The interleukin-1 receptor type 1 (IL1R1) gene was predicted as a DETG of LNC_003307 and was significantly increased in pig liver after the LPS challenge. Anzaghe et al. [47] have previously demonstrated that the organ-specific expression of IL1R1 treatment mediates poly(I:C)-induced IL-1β-mediated liver injury. In addition, the JAK2-STAT1/STAT3 signaling pathway plays an important role in initiating inflammatory response following LPS [48,49]. We predicted *JAK2*, *STAT1*, and *STAT3* were the DETGs identified for LNC_003307, suggesting LNC_003307 might function as a regulator of inflammation in LPS-induced liver injury. Additionally, we found that several upregulated DETGs of LNC_003307 are involved in ERS response, such as Homocysteine-inducible endoplasmic reticulum stress-inducible ubiquitin-like domain member 1 (*Herpud1*), which is induced by ERS [50]. We found a markedly increased expression of *Herpud1* in septic pig liver, suggesting LPS might induce ERS. Furthermore, the cytoskeleton-associated protein 4 (CKAP4) is responsible for stabilizing ER structure [51]; the heat shock protein A member 5 (HSPA5) is widely present in the ER as a key regulator of ERS response [52], being significantly upregulated in the pig liver after LPS challenge; the Hypoxia-upregulated 1 (HYOU1) is a chaperone protein located in ER whose expression is upregulated in ERS-related diseases [53]; and calreticulin (CALR) is an ER-resident protein whose overexpression can stimulate transcriptional activity of NF-κB [54]. In our study, we found that LPS-induced *CALR* expression in the liver might stimulate NF-κB activation leading to aggravation of the inflammation. Hence, it is possible that LNC_003307 participates in ERS regulation during septic liver injury.

## 5. Conclusions

The expression profiles of lncRNAs and mRNAs and their potential biological functions were presented here for the first time, using liver tissues of LPS-challenged septic pigs. Our study provides deep insights into transcriptional differences in pig liver after the LPS challenge. It enhances the understanding of the physiological functions and the underlying mechanisms of lncRNAs involvement in septic liver injury.

## Figures and Tables

**Table 1:** Sequencing data from porcine liver tissue.

Sample Name	Raw Reads	Q30 (%)	Clean Reads	Clean Ratio (%)	Mapped Reads	Mapped Ratio (%)
N1	103,431,076	95.15	101,547,492	98.18	84,293,797	83.01
N2	153,426,742	95.37	150,724,416	98.24	124,265,319	82.45
N3	112,256,790	95.17	110,219,924	98.19	90,385,080	82.00
L1	130,247,426	95.28	127,916,070	98.21	103,566,992	80.96
L2	164,291,314	95.24	161,346,466	98.21	129,899,294	80.51
L3	139,897,100	94.90	137,305,488	98.15	113,383,824	82.58

N1, N2, and N3 represent the three control libraries (saline treatment); L1, L2, and L3 represent the three experimental libraries (LPS treatment).

**Table 2:** Top five DE lncRNAs expressed in pig livers.

DE lncRNAs	Liver_LPS FPKM	Liver_Control FPKM	log2(FC)	*q*-Value
LNC_003307	272.26	61.8278	2.13866	0.000249496
LNC_002814	15.5746	37.2039	−1.25626	0.000249496
LNC_002495	13.6656	32.3223	−1.24198	0.000480016
LNC_001034	6.56286	25.9666	−1.98426	0.0159756
LNC_000416	4.60465	21.7599	−2.24051	0.000249496

## Data Availability

All the RNA-seq data have been deposited in the NCBI database with accession number PRJNA908120. Our SRA records will be accessible with the following link after the indicated release date: https://www.ncbi.nlm.nih.gov/sra/PRJNA908120 (accessed on 1 March 2023). The data that support the findings of this study are available from the corresponding author upon reasonable request.

## Supplementary Materials

The following supporting information can be downloaded at: https://www.mdpi.com/article/10.3390/genes14040945/s1, Supplementary Table S1. Primer sequences used in qRT-PCR and SMARTer RACE; Supplementary Table S2. The DE lncRNAs and DE mRNAs in porcine liver tissues after LPS challenge; Supplementary Table S3. The *cis-* and *trans*-targets of the DE mRNAs; Supplementary File S1. Sequence of a transcript of LNC_003307.
