# Supplementary material for: RNA-Sequencing Characterization of lncRNA and mRNA Functions in Septic Pig Liver Injury
Source: Genes (Basel). 2023 Apr 20;14(4):945. doi: 10.3390/genes14040945 (PMC10137529; doi:10.3390/genes14040945)
Supplement: Supplementary file 1 [file genes-14-00945-s001.zip › Supplementary File S1.pdf]

File S1. Sequence of a transcript of LNC_003307

ACATGGGGAATTACTGAAAGCCAGAGACTCAGGCAAAGAGTTTCTGCTTCTGGTGGA
GAGGAAAGGAGGGCAGTGGACCTCAGGGGACTCAAGAGGGAGACACAATGGAATCT
GGCTTCACCTCCAAGGATGCTTATCTGAGCCATTTTAACCCTCAGGATTACCTAGAAAA
ATATTACAACTTTGGGGCCAAACACTCCGCAGAAGACCAGATTCTTAGGCATCTTCTGA
AAATCCTTTTCAAGATATTTTGCCTAGATGGTGTGAAGGGGGACCTCCTGATTGACATC
GGCTCTGGCCCCACCATCTATCAGCTCCTCTCTGCTTGTGAGTCTTTCAAGGAGATCAT
TGCCACCGACTACACGGACCAGAACCTGCAGGAGCTGGAGAAGTGGCTGAAGAAGG
AGCCAGGGGCCTTTGACTGGTCCCCAGTGGTGACCTATGTGTGTGAGCTCGAAGGGAA
CAGGTAGAGAAACTGGGGTTGGCCTTCTGGCTTTCTGAGGGTACCTGGGTGCCGGTTC
ATTTTTCAGAACCAGGAATTATCCTTAGAGTCCAAGGTGAAACTCAGAGGAAGCAGTG
TGTGTGTGCATAAGATTTTTCTAGCAATTATATATTTTAACATATCTATTATTTCATAATGAT
ATATTACAATGATGTAATTCCTTTAACTCACTCTTCTGCCCCTTATGCACTTGCATTTTAC
ACATTTACCTATATATAATCTATATATACTACAACACTAGAATACGTTGTTATTATTTTTGTT
TTAAACTGTCAGTAATGCTTTCGTTTTTGTTGTTTTATTTTTAATTGTGGTTAAAAGAATA
CATATAAAATTTATCATCTTAACTTTTTTTAAGTGTACAGTTTAGTAGTTTTAAATATATTA
ATATCAGTCTGTAATTTTCAACAAATATATATATTTGCATTATGTATTATGTATACATGCATT
ATGTATGTGTGTGTGTGTATAATCTTCTGTATTTATCCATATATCTGTCCTGTCTAATATTC
TTCATTCCTTTCTGCAGATCTAGGCTTCTATCTGGAATCATTTCTCTTCACCTGGAGAAA
TTCTTTCAGCATTTCGTTCTTTCTTTTTTATTTTTTGCTTTTCAGGGCCAAACCTGAAGTT
CCTGGCTAGGGGTTGAACTGGAGCTGCCGCTGCTGACCTATGCCACAGCCACAGCAAC
TCGAAATCCCAGCCACATCCATGCCCTACACCACAGCTCATAGCAATGCCAGATCCTTA
ACTCACTGAGCAGGGCCAGGGATCAAGCCCGTGTCCTCATGGATACTAGCATCGTTCAT
TAGCACTGAGCCACAAGGGGAACTCCATCTTTCTTTTTTTTCTTTTCTTTTCTTTTGTCT
TTTTTAGGGCCACACCCGCAGCATATAGAGGTTCCCAGGCTAGGGGTTGAATCGGAAC
TGTAGCTGCCGGCTTATGCCGCAGCCACAGCAACTCAGGATCTGAGCCATATCTGTGAC
CCACACCACAGCTCAGTGGGATCCTTAACCCACTGAGAGTGGCCAGGGATCGAACCTA
CATCCTCATAGACACTATGTCGAGTCCTTAACCCATGAGCCACAACTGGAACCCTTCAT
ATGCCATTTCAACCTGTAAATGCCTTACTTGGAATTTTATGCTGGCTATTGCCTTCAGAC
TGAAGATACGGTCTTCAAAATAAAAGTAATTTCAAATTCTTTTCTTGACAATGTGATTTC
ACAGAATGAAATTGATATTAACTAGAAAAAAACTGTAGCTATGAGGACTCAGACAATAT
CCAAGAGTATTAAATAAATAAGGAATTTCCCAGGGTTTATAGAATTTCAGGGAAAGCTT
ATAAAAAAAGTTCTTAGCCATTATCAGACAACACATCTTTCCAAAGTCCGAATCATGTG
TCAAGAAGCCTTTTAGAACTTTGTTCTCAATTTCCCTAGCTATTACAAACCTTTCCATCT
CCTTCAGAAACGAACCCTTAAAATCTGGCAGGATACTAAAACCATTACTTATCTTAAGC
CGGAATCATCGGCTTGTAACAATTGGCTCCAAAGTCACTGTCCCTTCCCAATCACTCAG
TTAGACTAAGGAACTGTATTGGGGGCAGTAAAATTCTGCATTCTCACCTTTATCCTCCTA
CCAGATCCTGTGGCTTAAACTATTTCGGGGATGATAGTGATCCCATTCAACTTAGGTCTT
TCTTGCATCTTCAAGTGCCTCTATCAACCCACAACCTATTTCCCCTGAGATTATTTCTTC
TTTATCTGAAGCTATTTCTTGCGCTGACTGCTGCCAAAGACAATTGGAGCAGACTCCTG
TTCCTCTGCCTTGGGAGAGTGACACAGACATCTAGACACCACGCATCTTCACAATTCTC
CGAAAAGCTGTAAGACACTGGCACCAAATTCTTGCCTCACACTCTTCATTTATCAAGTC
AGAAAGTCAAAGACGAAGCTATAACTAACAACTCAAGCATGAGCCAAGGGAAATCAG
CCTTTGCCTTAAGACATCCTAGGAACGGCATTCATTAACCTCTGGCTCCTGGAACTTGG

AACCTGTTGCCTTGTGGTCCCCAGTCTAGCAGCTTCCATATCATCTGGAAACTTGTTAG
GCATGCAGACTCTCAGGCTTACCTACAGAATCAGAATCTGCATTTTAATGACATCTGCA
GGGGACTGCGTGCATTTTACACATTTACCGAGGACCTATGTCTGGAACAGAGTTTCCCA
ACAGTCATGCCAGGACACGCTGGTGGGTAGGGATGAATTACAGGTGTGCCCTGCAGGT
ACTCCTTGGCCCTCAGGGCAGCCAGGAGGGGTCGGGGGAAGCTGGAGCGCCTCCTGG
CTGCCTCTATGTAAGAGCGGCTCCTCTGCTTACCTCACTGTACTTGACAGTGAGCCTGA
AGAGGTGGAGCTTCTCTGAAGTAAGTGCTCGTCATACAAAGATCTTTTGTATCTGTTTC
AAAAGATGGAGTTCTCCTGTGGCGCAGCGGCTTAAGGATCCAGCGTTGTCCCTGCAGC
AGCTCAGGTGGCGGCTGTGGTGAGGTTTGATCCCTGGCCCAGGAACTTCCATAAGCCA
AGGGCGTGGCCAGAAAAAAATTCATCTATTGCCCTCAATAGACACAGTTTTGCCAAATT
TGCTCCATGAGCTAGTGACCTGCCTCTATGCCAAAGCATCTGGGTCTTTTCCTGTCTTAT
GAAAGACTGACATTCTTTCTCAAAATCAGGATCTTAATGGTCATGATGATTGTCTCAGG
GACACCTAGACAAAGCTTCTACCTTTTAGTAAGCTGCCTTCTGTCTTGAAAAGGGGCT
CTCACTGTATTATCTCTTGGGGAGAATGAACAGGACAGCCGGAAAAACCTTCAACTGG
CCATTTCTTTCAAACTGTAAACATTTCTACTTGGGTGTCTCAAAGCATCTCAAGCTCAA
TGTGCCCAAAATAGAGCTAGGACCTTCCCCAAAAAAAAAAAAAAAAAAAAAAAAAA
AAAA
