# Supplementary material for: RNA-Sequencing Characterization of lncRNA and mRNA Functions in Septic Pig Liver Injury
Source: Genes (Basel). 2023 Apr 20;14(4):945. doi: 10.3390/genes14040945 (PMC10137529; doi:10.3390/genes14040945)
Supplement: Supplementary file 1 [file genes-14-00945-s001.zip › Supplementary Table S1.pdf]

Table S1. Primer sequences used in qRT-PCR and SMARTer RACE.

Gene	Sequence (5′-3′)
LNC_001557	F:GAGGCTCGGGAGTCGTTT<br>R:CATTTACATAACCCGCTGGC
LNC_003205	F:AGTGGACCCAGAAAGACAGTAGT<br>R:GTGAGACATGGAATTGGAGAGAT
LNC_002154	F:CTAAGACAGCCGTCCATCCA<br>R:GGTTCAAGCCTCAACAGTTCC
LNC_002153	F:TAGGGTCAGAATCTGGAGCG<br>R:CAGGAACCTCCACAGGCTCT
LNC_003307	F:ACTGAGTCAAGCCTCCCGA<br>R:CGAATGCTTGCAGTCGCT
LNC_000402	F:CTGGACCATAACTCAAGATTCG<br>R:CCTTTGCTTTATTTGTCGCC
LNC_000482	F:CATCTGGGTTCCTTGCTCG<br>R:TGTCTTGGCTTTTCCTGTCTG
LNC_000421	F:GAGGAGCAATGGACTGAACA<br>R:ACTGATGGGCAGAGGGAC
LNC_003317	F:GGAAGAAGGGACAACGCTG<br>R:ATTCCCCACCCGCTCTTTA
LNC_003319	F:TCAAACAACAACGGCTGACAT<br>R:CACCTCTGAAAATGGGGACA
LNC_003307 5′ RACE GSP	GATTACGCCAAGCTTATTCATCCCTACCCACCAGCGTGTCCT
LNC_003307 3′ RACE GSP (External)	GATTACGCCAAGCTTGCCTTGTGGTCCCCAGTCTAGCAGC
LNC_003307 3′ RACE GSP (Internal)	GATTACGCCAAGCTTAGGACACGCTGGTGGGTAGGGATGAA
